# HIV seroconcordance among heterosexual couples in rural KwaZulu‐Natal, South Africa: a population‐based analysis

**DOI:** 10.1002/jia2.25432

**Published:** 2020-01-08

**Authors:** Hae‐Young Kim, Guy Harling, Alain Vandormael, Andrew Tomita, Diego F Cuadros, Till Bärnighausen, Frank Tanser

**Affiliations:** ^1^ Africa Health Research Institute KwaZulu‐Natal South Africa; ^2^ KwaZulu‐Natal Research Innovation and Sequencing Platform (KRISP) KwaZulu‐Natal South Africa; ^3^ Department of Population Health New York University Grossman School of Medicine New York NY USA; ^4^ Institute for Global Health University College London London UK; ^5^ Department of Epidemiology & Harvard Center for Population and Development Studies Harvard T.H. Chan School of Public Health Boston MA USA; ^6^ MRC/Wits Rural Public Health & Health Transitions Research Unit (Agincourt) University of the Witwatersrand Johannesburg South Africa; ^7^ School of Nursing and Public Health University of KwaZulu‐Natal Durban South Africa; ^8^ Heidelberg Institute of Global Health Faculty of Medicine University of Heidelberg Heidelberg Germany; ^9^ Centre for Rural Health School of Nursing and Public Health University of KwaZulu‐Natal Durban South Africa; ^10^ Department of Geography and Geographic Information Science University of Cincinnati Cincinnati OH USA; ^11^ Department of Global Health and Population Harvard T.H. Chan School of Public Health Boston MA USA; ^12^ Lincoln Institute for Health University of Lincoln Lincoln UK; ^13^ Centre for the AIDS Programme of Research in South Africa (CAPRISA) University of KwaZulu‐Natal KwaZulu‐Natal South Africa

**Keywords:** HIV, seroconcordance, serosorting, heterosexual couples, sexual partnership, assortative sexual mixing, South Africa

## Abstract

**Introduction:**

High levels of HIV seroconcordance at the population level reduce the potential for effective HIV transmission. However, the level of HIV seroconcordance is largely unknown among heterosexual couples in sub‐Saharan Africa. We aimed to quantify the population level HIV seroconcordance in stable heterosexual couples in rural South Africa.

**Methods:**

We followed adults (≥15 years old) using a population‐based, longitudinal and open surveillance system in KwaZulu‐Natal, South Africa, from 2003 to 2016. Sexual partnerships and HIV status were confirmed via household surveys and annual HIV surveillance. We calculated the proportions of HIV seroconcordance and serodiscordance in stable sexual partnerships and compared them to the expected proportions under the assumption of random mixing using individual‐based microsimulation models. Among unpartnered individuals, we estimated the incidence rates and hazard of sexual partnership formation with HIV‐positive or HIV‐negative partners by participants' own time‐varying HIV status. Competing risks survival regressions were fitted adjusting for sociodemographic and clinical factors. We also calculated Newman's assortativity coefficients.

**Results:**

A total of 18,341 HIV‐negative and 11,361 HIV‐positive individuals contributed 154,469 person‐years (PY) of follow‐up. Overall, 28% of the participants were in stable sexual partnerships. Of the 677 newly formed stable sexual partnerships, 7.7% (95% CI: 5.8 to 10.0) were HIV‐positive seroconcordant (i.e. both individuals in the partnership were HIV‐positive), which was three times higher than the expected proportion (2.3%) in microsimulation models based on random mixing. The incidence rates of sexual partnership formation were 0.54/1000PY with HIV‐positive, 1.12/1000PY with HIV‐negative and 2.65/1000PY with unknown serostatus partners. HIV‐positive individuals had 2.39 (95% CI: 1.43 to 3.99) times higher hazard of forming a sexual partnership with an HIV‐positive partner than did HIV‐negative individuals after adjusting for age, opposite‐sex HIV prevalence (by 5‐years age groups), HIV prevalence in the surrounding community, ART coverage and other sociodemographic factors. Similarly, forming a sexual partnership with an HIV‐negative partner was 1.47 (95% CI: 1.01 to 2.14) times higher in HIV‐negative individuals in the adjusted model. Newman's coefficient also showed that assortativity by participant and partner HIV status was moderate (r = 0.35).

**Conclusions:**

A high degree of population level HIV seroconcordance (both positive and negative) was observed at the time of forming new sexual partnerships. Understanding factors driving these patterns may help the development of strategies to bring the HIV epidemic under control.

## 1 Introduction

It is estimated that about one out of five adults in South Africa were living with HIV in 2018 1, 2. In sub‐Saharan Africa (SSA), there is increasing evidence that HIV transmission within married or cohabiting couples is a major factor driving the generalized HIV epidemic 3, 4, 5. A recent study in South Africa showed that having an HIV‐positive cohabiting partner not on ART increased the risk of HIV acquisition for the uninfected partner by almost two‐fold compared to being in a non‐cohabiting relationship 4. In rural Zambia, most heterosexual HIV transmission occurred within marriage or cohabitation 3. HIV serostatus at the time of stable partnership formation could play an important role on HIV transmission at the individual and population level in generalized HIV epidemic settings.

Past studies have shown that men‐who‐have‐sex‐with‐men (MSM) are partnering with individuals of the same HIV serostatus (i.e. seroconcordance), which was adapted as a strategy to potentially reduce HIV transmission in a stable and non‐stable relationship 6, 7, 8, 9, 10. For example, modelling studies have shown that serosorting by perceived HIV status, combined with status‐based condom use, provided modest protection from HIV acquisition and contributed to reduced HIV transmission among the MSM population in the United States 11. Research on the protective benefits of HIV serosorting among MSM (i.e. intentional seroconcordance) has, however, been mostly limited to high‐income settings 12, 13. Despite the clear evidence of serosorting in MSM, evidence on seroconcordance and serosorting in heterosexual couples is largely unknown, especially in resource‐limited settings in SSA.

Some qualitative evidence suggests that serosorting occurs and has substantial benefits in heterosexual couples. HIV‐positive seroconcordant couples report having increased bonding and support for each other, resulting in better healthcare access and treatment adherence 14, 15. A qualitative study in South Africa reported that women living with HIV might seek HIV‐positive partners, who could share the same fertility goals 16. At the population level, modelling studies which incorporated the degree of preferential partnership formation (i.e. assortative mixing) predicted lower HIV incidence rates if individuals preferred partners with similar sexual behaviour risks 17, 18. However, to the best of our knowledge, no study has quantified the degree of seroconcordance in heterosexual couples at the population level in SSA and compared it to the expected levels of seroconcordance for a given HIV prevalence.

The Africa Health Research Institute (AHRI) has maintained an open population‐based surveillance system, undertaken annual HIV testing, and collected detailed information on household memberships and sexual partnerships since 2003 19. This comprehensive and detailed data provides a unique opportunity to examine factors associated with the formation of new stable sexual partnerships among over 30,000 sexually active adults with known HIV status. The objective of this study was to quantify the level of HIV seroconcordance and serodiscordance in stable heterosexual couples in rural South Africa. First, we calculated the proportions of HIV seroconcordance and serodiscordance in the reported stable sexual partnerships in heterosexual couples. Second, among individuals who were unpartnered, we quantified the incidence rates and hazard of HIV seroconcordance and serodiscordance in newly formed stable sexual partnerships. Understanding factors driving these patterns will help to investigate the impact of seroroconcordance on HIV incidence and transmission at the population level and develop strategies to improve epidemic control.

## 2 Methods

### 2.1 Study setting and procedures

We used the data from a population‐based surveillance system in a rural part of the uMkhanyakude district, KwaZulu‐Natal, South Africa 19. The surveillance site covers 438 km^2^ in size and includes over 100,000 individuals and 11,000 households. All individuals who are members of households in the surveillance area are enrolled into the demographic surveillance system. Households are defined as social groups of individuals who largely share the same resources, have one household head, know basic information about each other, and members can be either residents or non‐residents 19. Trained field‐workers visit all households in the surveillance area and interview a key informant every four to six months. The key informant is often the household head or the most senior household member and provides information on demographic attributes of household members including composition, migration events, mortality and marital status.

In the surveillance area, the marriage rate is low at <10% among men and women aged 20 to 45 years 20, 21. Thus, detailed information is also collected on stable sexual relationships among heterosexual couples which include both marital and non‐marital relationships. We defined the relationships as “stable” if the partners in these relationships progressed to belonging to the same household (i.e. conjugal relationships), in constrast to casual partnerships which are often transient and the partners do not become the same household members. Stable sexual partners may or may not cohabit in the same residence 22. Information on stable sexual relationships and partners including the start and end dates of the relationships is only sought from female household members, but male partners are linked with female partners via the demographic surveillance system. From this linkage, we were able to determine the partners’ HIV status. The overall household response rates are >95% 23.

Nested within the demographic surveillance is an annual HIV survey. After obtaining written informed consent, resident individuals aged 15 years or older provide dried blood spots for HIV testing and complete a sexual behaviour survey 24. The sexual behaviour survey includes self‐reporting of the number of current sexual relationships and sexual partners in the past 12 months.

### 2.2 Study participants

All adults (≥15 years old) enrolled in the demographic surveillance were eligible if they had a first HIV‐negative test result followed by at least one more HIV test, or had a first HIV‐positive test. All eligible individuals were included in the analysis to measure the proportions of the current stable sexual partnerships. To estimate the rates of new sexual partnership formation, only individuals who were unpartnered (i.e. not in any stable sexual partnership) were included in the analysis and followed up from the earliest HIV‐negative or HIV‐positive test date until the formation of stable sexual partnership. We assumed that an individual can only transition from being unpartnered to being in a stable sexual partnership and vice versa (i.e. cannot be in concurrent stable sexual partnerships).

### 2.3 Exposures

The primary exposure was each participant's own time‐varying HIV status, ascertained from the annual HIV survey. For both participants and partners who seroconverted during the follow‐up period, HIV status was interval censored where the mid‐point between the date of last HIV‐negative test and the date of first HIV‐positive test was used as a proxy for the date of HIV infection to correctly attribute the exposure time of HIV status 25. HIV status was censored on the last HIV‐negative test date for those who never tested positive.

Partner's HIV status was considered as positive if the stable sexual partnership was formed after the partner's first HIV‐positive test or seroconversion date; or negative if formed before the partner's last HIV‐negative test or seroconversion date. Partner's HIV status was considered as unknown if the partner's HIV test result was unavailable or unknown at the time of the partnership formation. We performed a sensitivity analysis where we imputed a random date of seroconversion from a uniform distribution bounded by the latest‐negative and earliest‐positive test dates 26 (Table S4 and Figure S4).

### 2.4 Outcomes

The primary outcome was the level of HIV seroconcordance and serodiscordance in the current and new stable sexual partnerships. First, we calculated the proportion of participants who were in stable sexual partnerships and then measured the degree of HIV seroconcordance and serodiscordance in the reported stable sexual partnerships in each year. Second, among those who were unpartnered, we estimated the incidence rates for stable sexual partnership formation, allowing only one partnership formation per participant. We also calculated the proportions and hazard of HIV seroconcordance and serodiscordance in new stable sexual partnerships. Participants who formed stable sexual partnerships were right‐censored on the start dates of the stable sexual partnerships. Those who had not formed a partnership were censored on the last date of a household visit when the information on stable sexual partnership status was collected. Individuals already in stable sexual partnerships were included in the analysis once they dissolved previous partnerships.

### 2.5 Covariates

We considered age at baseline as a fixed binary covariate (aged <30 vs. ≥30) and completed education, area of residence and household wealth (quantiles of the leading component of a principal components analysis) as time‐varying covariates 27. We also included HIV prevalence in the opposite sex among adults in the same age group (15 to 19, 20 to 24, 25 to 29, 30 to 34, 35 to 39, 40 to 44, 45 to 49, 50+ years) in each calendar year, since the probability of selecting an HIV‐positive heterosexual partner depends on the HIV prevalence of the opposite sex 24. We also included two time‐varying geographic measures – HIV prevalence and ART coverage – based on the household location in each calendar year using Gaussian kernel weights of search radius 3 km 25. If individuals resided in multiple locations within a year, residence was defined by where they spent most days. About 16% of the data for household wealth were missing while other covariates had <2% missingness. Sexual behaviour surveys were prone to high incompletion or refusal rates in any given survey round 24. Thus, we fitted ever reporting more than one sexual partner in the past 12 months during the follow‐up period as a fixed binary covariate, which had about 13% missing data.

### 2.6 Statistical analysis

We used descriptive analysis to calculate the proportions of HIV seroconcordance and serodiscordance in the reported stable sexual partnerships in each year. For new sexual partnership formation, we performed competing‐risks survival regression to estimate the incidence rates and the hazard of stable sexual partnership formation with known HIV seropositive partners by participants' HIV status, where formation with known seronegative or unknown serostatus partners was fitted as the competing risks using the Fine and Gray's subhazard model 28. Similarly, we also estimated the hazard of stable sexual partnership formation with known HIV seronegative partners, where formation with known seropositive or unknown serostatus partners was fitted as the competing risks. The proportionality hazard assumption was checked by fitting interaction terms between covariates and time. Time to event was defined from the first HIV test date with known test results to the start date of stable sexual partnership or the censored date for those who never formed stable sexual partnerships during the follow‐up. Although the formation of a stable sexual partnership was only ascertained from female household members, we included the time to partnership formation from both female and male participants, assuming that males independently decide to form stable sexual partnerships with female partners. Any missing data for covariates were fitted as unknown in the adjusted models. We have also run sensitivity analysis to handle the missing data for covariates using multiple imputation (See supplement). We additionally performed the analyses stratified by females and males. Hazard ratios (HR) and 95% confidence intervals (CI) are reported.

We calculated Newman's assortativity coefficients for partnership formation by participants' and partners' HIV status, ranging from −1 to 1; positive coefficients represent assortativity while negative coefficients represent disassortativity 29. We have used the previously defined categories of assortativity in HIV seroconcordance among heterosexual couples where the assortativity coefficients ≥0.35 were considered as assortative, 0.15 to 0.34 as moderately assortative, and <0.15 as not indicative of assortativity (i.e. random mixing) 30, 31.

All analyses were conducted in STATA 15.1 (Statacorp; College Station, TX). Both demographic and HIV surveillance were approved by the University of KwaZulu‐Natal Biomedical Research Ethics Committee (BE290/16).

### 2.7 Individual‐based microsimulation model

We constructed a stochastic individual‐based microsimulation model to assess the expected proportions of HIV seroconcordance for stable sexual partnership formation assuming random mixing in a population and compared those to the observed proportions. The model was parameterized using the variables derived from the study population such as the total numbers of males and females, HIV prevalence per year by sex, proportions of known and unknown HIV status, and the total numbers of stable sexual partnership formation from 2003 to 2016. Results of the microsimulation are based on 10,000 realizations of the model. The model was developed using MATLAB R2016b. Details of model description and results are described in the supplementary materials.

## 3 Results

Between January 2003 and December 2016, 29,702 individuals contributing 154,469 person‐years (PY) follow‐up time met the inclusion criteria in the study. This comprised 18,341 (61.7%) always HIV‐negative and 11,361 (38.3%) ever HIV‐positive individuals. There were more females than males (18,939, 63.8%) and median age at baseline was 22 (Interquartile Range (IQR): 17 to 36) and over >80% had never been married (Table 1). On average, 28.3% of participants reported being in stable sexual partnerships in each year (Table 2). Of these partnerships, 11.9% were HIV‐positive seroconcordant (i.e. both individuals in the partnership were HIV‐positive) and 24.2% were HIV‐negative seroconcordant (i.e. both individuals in the partnership were HIV‐negative). Only 6.5% were HIV serodiscordant, while the remaining 57.3% of partnerships were with the partners of unknown serostatus. Thus, of the partnerships in which both partners HIV status were known, HIV‐positive and HIV‐negative seroconcordance was 27.9% and 56.7% respectively.

**Table 1:** Baseline characteristics of study participants in the surveillance area, KwaZulu‐Natal, South Africa (N = 29,702)

Characteristics	
Age at baseline (years)	
Median (Q1, Q3)	22 (17, 36)
Sex, n (%)	
Male	10,763 (36.2)
Female	18,939 (63.8)
HIV status, n (%)	
Negative	21,363 (71.9)
Positive	8339 (28.1)
Region, n (%)	
Rural	17,919 (62.8)
Peri‐urban	9189 (32.2)
Urban	1422 (5.0)
Asset quantiles, n (%)^a^	
Poorest	2826 (13.7)
Poor	4561 (22.1)
Medium	4746 (23.0)
Rich	4439 (21.5)
Richest	4071 (19.7)
Education, n (%)	
No formal education	2676 (9.6)
Primary (Grade 1 to 7)	8026 (28.9)
Secondary+ (Grade 8+)	17,078 (61.5)
Ever reporting to have more than one sexual partner in the last 12 months, n (%)^a^	
Yes	2206 (8.8)
No	22,770 (91.2)
Marriage, n (%)	
Married	995 (3.6)
Separated, divorced, or widowed	2644 (9.5)
Single	24,336 (87.0)

a Variable has missing values for more than 10% of the records.

**Table 2:** Proportion of stable sexual partnerships by HIV status of participants and their partners from 2003 to 2016

Year	N	Currently in stable sexual partnership, %(n)^a^	HIV‐negative participants^b^			HIV‐positive participants		
			Partner HIV status, %(n)			Partner HIV status, %(n)		
			Negative	Positive	Unknown	Negative	Positive	Unknown
2003	2852	31.7 (904)	21.3 (193)	1.9 (17)	72.5 (655)	0.4 (4)	0.8 (7)	3.1 (28)
2004	10,359	32.4 (3355)	21.1 (709)	2.4 (82)	53.4 (1790)	1.9 (65)	4.4 (147)	16.8 (562)
2005	14,292	30.0 (4290)	22.2 (951)	2.9 (126)	45.9 (1969)	2.7 (117)	6.6 (281)	19.7 (846)
2006	15,464	29.2 (4515)	22.1 (1000)	3.3 (150)	42.8 (1931)	3.3 (148)	8.6 (387)	19.9 (899)
2007	18,319	30.9 (5657)	25.9 (1465)	3.0 (169)	43.5 (2459)	2.8 (157)	8.0 (455)	16.8 (952)
2008	19,732	30.9 (6088)	27.2 (1658)	3.3 (201)	40.5 (2464)	3.2 (195)	9.0 (548)	16.8 (1022)
2009	19,703	30.7 (6045)	27.4 (1658)	3.3 (201)	38.6 (2333)	3.3 (198)	9.8 (594)	17.6 (1061)
2010	21,473	29.7 (6376)	26.9 (1714)	3.3 (208)	35.5 (2263)	3.4 (219)	11.8 (753)	19.1 (1219)
2011	21,017	29.3 (6166)	26.3 (1620)	3.4 (207)	34.4 (2122)	3.7 (230)	12.6 (774)	19.7 (1213)
2012	20,931	28.1 (5886)	25.7 (1513)	3.3 (195)	33.4 (1968)	3.7 (218)	13.5 (792)	20.4 (1200)
2013	21,273	27.1 (5761)	24.4 (1407)	3.5 (201)	32.0 (1845)	3.8 (220)	14.5 (833)	21.8 (1255)
2014	21,149	25.5 (5401)	23.7 (1281)	3.6 (193)	31.1 (1679)	3.9 (209)	15.6 (840)	22.2 (1199)
2015	20,603	24.2 (4932)	21.1 (1042)	3.3 (161)	30.6 (1509)	3.8 (188)	18.4 (909)	23.8 (1173)
2016	14,796	21.4 (3168)	12.3 (390)	1.6 (52)	18.6 (590)	5.0 (159)	27.4 (869)	35.0 (1108)
Annual average	17,283	28.3 (4896)	24.2 (1185)	3.1 (154)	37.3 (1826)	3.4 (166)	11.9 (584)	20.0 (981)

a Proportion is calculated as the number of people in stable sexual partnerships in the mid‐year (June 30th) of each calendar year among the total participants with known HIV status

b proportion is calculated as the number of stable sexual partnership with HIV‐negative, HIV‐positive, or unknown HIV status partners by the HIV status of the participants divided by the total number of people in stable sexual partnerships.

Overall, there were 677 stable sexual partnerships newly formed from 2003 to 2016. Of these, the great majority (99.3% (363/366) in females and 93.3% (290/311) in males) formed only one stable sexual relationship, or without concurrency if participants formed more than one stable sexual relationship. Overall, 59.3% (217/366) of females and 54.7% (170/311) of males completed the annual sexual health survey at least once while being in stable sexual partnerships. Among females, there was only one record (0.2%, 1/454) reporting being in more than one sexual relationship, and two records (0.3%, 2/585) reporting having more than one sexual partner in the past 12 months. Among males, 6.7% (17/254) reported being in more than one sexual relationship while 9.9% (36/363) had more than one sexual partner in the past 12 months.

Of the 677 new stable sexual partnerships, 7.7% (95% CI: 5.8 to 10.0) were HIV‐positive seroconcordant while 12.4% (95% CI: 10.0 to 15.1) were HIV serodiscordant. The proportion of HIV‐negative seroconcordant partnerships was 19.4% (95% CI: 16.4 to 22.5). Results from the microsimulation indicated that assuming random partnership selection, regardless of HIV status of the partner, and allowing for the same proportion of individuals with unknown HIV status to be partnered, only 2.3% (95% CI: 1.3 to 3.5) of the newly formed stable sexual partnerships would be HIV‐positive seroconcordant (more than three times lower than the percentage observed in the cohort), whereas 15.2% (95% CI: 12.5 to 17.8) would be HIV serodiscordant (Table S1). The model estimated a slightly higher (but not statistically different) proportion of HIV‐negative seroconcordant couples (25.1%; 95% CI: 21.9 to 28.3) than observed in the cohort (Figure S2).

**Figure 1:**
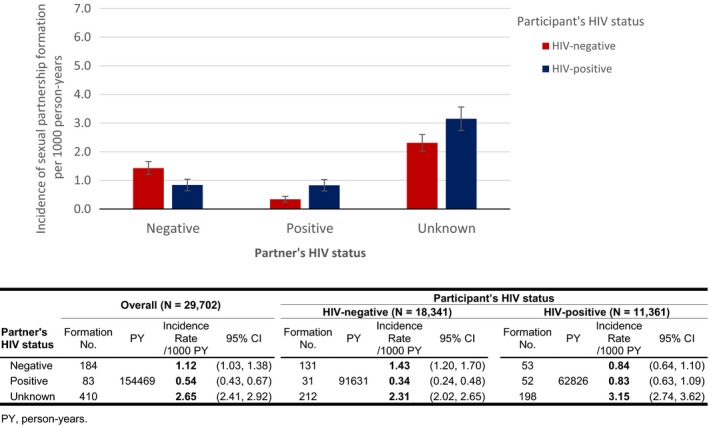
Incidence rates for stable sexual partnership formation per 1000 person‐years with an HIV‐positive, HIV‐negative or unknown serostatus partner by participant's own time‐varying HIV status. Error bars represent 95% confidence intervals for the incidence rates of stable sexual partnership formation per 1000 person‐years.

Incidence rates of stable sexual partnership formation by participants' own and partners' time‐varying HIV status are shown in Figure 1. We observed 677 (4.38 per 1000 PY) new stable sexual partnerships formations: 0.54/1000 PY with known HIV‐positive, 1.12/1000 PY with known HIV‐negative and 2.65/1000 PY with unknown serostatus partners. When we stratified by HIV status of participants, the incidence of stable sexual partnership formation with known seropositive partners was higher in HIV‐positive individuals than in HIV‐negative individuals (0.83/1000 PY vs. 0.34/1000 PY, *p* < 0.001). The incidence of stable sexual partnership formation with unknown serostatus partners was also higher among HIV‐positive individuals than among HIV‐negative individuals (3.15/1000 PY vs. 2.31/1000 PY, *p* < 0.001). When we stratified by participants' sex, we observed the similar patterns of stable sexual partnership in both men and women (Figure S3). Among participants partners with known HIV status, the overall Newman's coefficient for partnership formation was 0.35. The Newman's coefficient was slightly higher among men (0.40) than among women (0.28).

In unadjusted analysis, HIV‐positive individuals had a 2.83 (95% CI: 1.78 to 4.49) times higher hazard of forming a partnership with an HIV‐positive partner than did HIV‐negative individuals (Table 3). This was slightly attenuated after adjusting for covariates (Adjusted HR (AHR)=2.39, 95% CI: 1.43 to 3.99). We did not see any significant effect‐modification of the association by sex (Table 4). Forming a sexual partnership with an HIV‐negative partner was 1.47 (95% CI: 1.01 to 2.14) times higher in HIV‐negative individuals than in HIV‐positive individuals after adjusting for covariates (Table S2). There were no significant changes in the model estimates when missing covariates were adjusted using multiple imputation (Table S3).

**Table 3:** Hazard ratios for stable sexual partnership formation with HIV‐positive partners among all participants

Characteristics	Model 1	Model 2
	Hazard Ratio (95% CI)	Adjusted Hazard Ratio (95% CI)^a^
HIV status		
Positive versus Negative	**2.83 (1.78 to 4.49)** **	**2.39 (1.43 to 3.99)** **
HIV prevalence in the opposite sex (per 10% increase)		1.01 (1.00 to 1.03)
ART coverage (per 10% increase)		0.89 (0.74 to 1.06)
HIV prevalence in the local area (per 10% increase)		1.15 (0.72 to 1.85)
Age at baseline (years)		
≥30 versus <30		1.71 (0.96 to 3.02)
Socioeconomic status (Household Asset)		
Poorest or poor		**2.93 (1.49 to 5.78)** **
Medium		1.54 (0.67 to 3.55)
Rich or richest		Ref
Education		
Secondary+ (≥grade 8)		**0.43 (0.22 to 0.85)** *
Primary (grade 1 to 7)		0.54 (0.27 to 1.10)
No formal education		Ref
Area of residence		
Peri‐urban or urban versus Rural		1.64 (0.88 to 3.04)
Ever reporting to have >1 partner in the last 12 months		
Yes versus No		1.84 (0.90 to 3.74)

ART, antiretroviral therapy; HIV, human immunodeficiency virus.

a The model was adjusted for all other variables shown in the column.

*p‐value < 0.05; **p‐value < 0.01.

**Table 4:** Adjusted hazard ratios for stable sexual partnership formation with HIV‐positive partners among females (Model 3) and males (Model 4)^a^

Characteristics	Model 3 (female)	Model 4 (male)
	Adjusted hazard ratio (95% CI)	Adjusted hazard ratio (95% CI)
HIV status		
Positive versus Negative	**2.49 (1.16 to 5.31)** *	**2.18 (1.07 to 4.44)** *
HIV prevalence of the opposite sex (per 10% increase)	**1.03 (1.00 to 1.05)** *	0.99 (0.98 to 1.01)
ART coverage (per 10% increase)	0.78 (0.60 to 1.01)	1.05 (0.80 to 1.38)
HIV prevalence in the local area (per 10% increase)	0.82 (0.39 to 1.73)	1.59 (0.90 to 2.81)
Age at baseline (years)		
≥30 versus <30	0.80 (0.40 to 1.61)	**4.14 (1.65 to 10.40)** **
Socioeconomic status (Household Asset)		
Poorest or poor	3.13 (0.92 to 10.70)	**2.29 (1.00 to 5.26)** *
Medium	3.25 (0.90 to 11.82)	0.68 (0.18 to 2.51)
Rich or richest	Ref	Ref
Education		
Secondary+ (≥ grade 8)	**0.36 (0.14 to 0.92)** *	0.43 (0.16 to 1.12)
Primary (grade 1 to 7)	**0.33 (0.11 to 0.97)** *	0.72 (0.28 to 1.87)
No formal education	Ref	Ref
Area of residence		
Peri‐urban or urban versus Rural	1.53 (0.58 to 4.04)	1.79 (0.81 to 3.94)
Ever reporting to have >1 partner in the last 12 months		
Yes versus No	2.44 (0.74 to 8.02)	1.31 (0.55 to 3.10)

ART, antiretroviral therapy; HIV, human immunodeficiency virus.

a The model was adjusted for all other variables shown in the column.

*p‐value < 0.05; **p‐value < 0.01.

## 4 Discussion

To the best of our knowledge, this is the first study to quantify the population‐level HIV seroconcordance and serodiscordance in heterosexual couples in a high HIV prevalence and resource‐limited setting. Most previous reports on serocondcordance have focused on MSM in high‐income settings, who often adopt serosorting as a harm reduction strategy for HIV transmission and acquisition 6, 11, 32, 33. Heterosexual couples are likely to have different motivations for sexual partner selection than the MSM population 3, 5. Overall, we found that while lower levels of ART coverage or low socioeconomic status were independently associated with a higher hazard of HIV‐positive seroconcordant partnership formation, individuals living with HIV are two times more likely to form stable sexual partnerships with partners of the same HIV status, independent of age, HIV prevalence in the opposite sex, local HIV prevalence and ART coverage, and other sociodemographic characteristics. Such association was similarly observed for HIV‐negative seroconcordance, although the degree was stronger for HIV‐positive seroconcordance. The observed level of HIV‐positive seroconcordance was more than three times higher than the estimated level using microsimulation assuming random sexual mixing. In addition, the Newman's assortativity coefficient also showed moderately high assortativity by participants' and partners' HIV status. Altogether, these results suggest the presence of structured partner selection within the study population when heterosexual couples seek and form partnerships in this rural hyperendemic setting.

One potential explanation for higher HIV seroconcordance among HIV‐positive heterosexual couples is that individuals living with HIV may have intentionally chosen partners with the same HIV status. In high‐income settings, several quantitative and qualitative studies have reported that MSM choose seroconcordant partners to reduce the risk of HIV acquisition and transmission 6, 7, 8, 9, 10, 13, and more likely to do so than heterosexual men and women 34. Evidence from heterosexual couples in SSA suggests that individuals living with HIV may seek and receive more social support from seroconcordant partners 14, 15. In our study setting, being in a conjugal relationship is associated with more social connection and better health outcomes, including lower rates of all‐cause mortality, which suggests that social support flows through conjugal relationships, and may flow more strongly in seroconcordant ones 35.

Sexual partners are often the first person with whom an individual shares their HIV‐positive status, but also the person whose response upon disclosure they most fear 36, 37. Females might be particularly vulnerable upon disclosure of HIV‐positive status, potentially experiencing intimate partner violence, dissolution of relationships or desertion from partners due to their HIV status 38, 39, 40. An HIV‐positive partner might reduce the stress of disclosing HIV status 16 and ease condom use negotiation 41. As a result, HIV‐positive women may prefer seroconcordant partners when committing to new long‐term relationships. It is possible that individuals with a high risk of HIV were likely to select partners who had a comparable risk for HIV infection such as living in an area with higher HIV prevalence. However, after adjusting for other factors associated with the risk of HIV, seroconcordant partnering was still much higher than serodiscordant partnering. Both longitudinal quantitative and qualitative studies could help to elaborate on the mechanisms for seroconcordance, and the reasons behind potential serosorting decisions, as well as to examine the psychosocial and clinical impact of serosorting, and to measure the association of seroconcordance with long‐term health outcomes among HIV‐positive individuals.

Some critical questions remain regarding the impact of HIV serosorconcordance in generalized heterosexual HIV epidemics. These include understanding how partner seroconcordance impacts HIV incidence at the population level: a recent modelling study showed that selecting partners based on ART (“ART homophily”) can potentially reduce HIV transmission in hyperendemic settings when the rate of ART coverage is high but ART adherence remains low 42. Other modelling studies have shown that preferential partnership with those in the same risk groups could result in reduction of HIV incidence rates by half 17, 43. However, these models defined risk groups based on engaging in commercial sex or in concurrent sexual relationships, not based on individuals’ HIV status 43. Our study provides the first population‐level evidence that “preferential mixing” by individuals’ own and partners’ HIV status occurs in heterosexual couples in hyper‐endemic settings. Modelling of how much such preferential mixing might impact the overall epidemic would be instructive to design interventions for the epidemic control.

Other factors may affect the association between seroconcordance and the risk of HIV transmission. In both MSM and heterosexual women, serosorting was associated with risk compensation in the form of increased condomless sex, making the benefits of serosorting greatly dependent on the accuracy of partners HIV status perception and other behavioural strategies 44, 45, 46, 47, 48. At the same time, among MSM, HIV‐negative MSM who self‐report as practicing serosorting are estimated to have a 54% lower risk of HIV acquisition, compared to condomless anal sex with either HIV‐positive or unknown status partners 13. With expansion of pre‐exposure prophylaxis (PrEP) globally, potential impact and role of serosorting behaviour might change as well. A recent study among young MSM in the US reported how access to and use of PrEP have shifted the paradigm about serosorting and made them open and neutral to “seromixing” and serodiscordance in seeking relationships 49. Future studies could investigate the effect of serosorting and related risk compensation on HIV incidence and ART uptake among heterosexual couples 50.

The biggest strength of our analysis is using the population‐based longitudinal dataset over 30,000 individuals for more than a decade, which allowed us to examine the new sexual partnership formation rates by both participants' and partners' HIV status. Since HIV prevalence in the opposite sex differs by age and would affect the likelihood of choosing HIV‐positive partners by random chance, we accounted for the large variation in underlying pools of HIV‐positive individuals by adjusting for the HIV prevalence in the opposite sex and in the local boundary area estimated in the study setting 25, 51, 52. Another strength we used is the concept of stable sexual partnership, which is a better reflection of a regular sexual partnership than marital status. Most studies use marital status as the indicator of regular partnerships in heterosexual couples. However, in the surveillance area, only 10% of women aged 20 to 45 years were currently married in 2009 while more than half of men and women aged <39 years experienced at least one formation of a conjugal relationship 20, 21, 53. Lastly, the proportions of concurrent stable partnerships or causal sexual partnerships were quite low among those in stable partnerships. Thus, the role of seroconcordance and serodiscordance in stable couples seems important for HIV prevention strategies.

There are several limitations to our study. First, a high proportion (>50%) of individuals had partners with unknown HIV status. To adjust potential bias, we included formation with a partner with unknown HIV status as a competing risk in the analysis. Also, we simulated the models including the proportion of partners with unknown HIV status and still found a much higher level of HIV‐positive seroconcordance than expected. Second, our analyses may have suffered from residual confounding if we have not captured some factors that affect partner selection in this setting, given that long‐term partner selection is affected by a wide range of structural, interpersonal and psychological factors. Third, we only considered seroconcordance within stable relationships but not in casual partnerships. This reflected the difficulty of ascertaining casual partners’ HIV status and our expectation that the processes of partner selection and HIV disclosure in casual sexual partnerships could be systematically different from that seen in regular sexual partnerships. We nevertheless adjusted for the number of sexual partners in the past 12 months as an indicator of sexual engagement with casual partners. Also, we ascertained HIV status once the couples have become stable relationships. It is possible that seroconcordant couples were more likely to have progressed to stable relationships than serodiscordant couples given the difficulties introduced by disclosure of HIV status in serodiscordant couples, thus differentially included in our analysis. Nevertheless, this would not undermine the implication of our findings on the population‐level epidemics and potential behavioural health outcomes among stable relationships. Further studies to determine whether prospective couples know and share their HIV status before forming sexual relationships or during the transition to stable relationships could be instructive. Lastly, the simulation model was simple but the purpose of the model was to demonstrate random mixing regardless of HIV status. While the current model well supported our finding of the high level of seroconcordance, further work would be needed to incorporate other characteristics related to partner selection and formation.

## 5 Conclusions

In this study, we quantified the high degree of seroconcordance and found that patterns of partnership formation are not random in this HIV endemic setting. HIV‐positive individuals were more likely to initiate stable conjugal relationships with partners who were also HIV‐positive, and HIV‐negative individuals with HIV‐negative ones. We have therefore built a platform for future work to understand the implications of seroconcordance for HIV incidence and transmission at the population level, and to explore intervention options that can leverage seroconcordance to promote better long‐term health outcomes.

## Competing interests

All authors have no competing interests to report.

## Authors' contributions

H‐YK, GH, TB and FT conceptualized and designed the study. H‐YK performed the literature search, acquired the data, ran the analyses and wrote the initial draft. DC ran the microsimulation models. FT helped to acquire the data and provided overall supervision for data analysis and interpretation. All authors contributed to interpret the results, read and revised the final manuscript.

## Supporting information


**Data S1.** Individual‐based microsimulation model description.
**Table S1.** Estimated proportion of each type of the partnerships. Estimations are based on 10,000 realizations of the model.
**Table S2.** Hazard ratios for stable sexual partnership formation with HIV‐negative partners among all participants.
**Table S3.** Hazard ratios for stable sexual partnership formation with HIV‐positive partners among all participants.
**Table S4.** Estimated proportion of each type of the partnerships using the random impuation method for HIV seroconversion dates. Estimations are based on 200 realizations of the model.
**Figure S1.** Distribution of the proportion of HIV‐positive seroconcordant partnerships. Estimations are based on 10,000 realizations of the model.
**Figure S2.** Estimated proportion of each type of the partnerships. Estimations are based on 10,000 realizations of the model.
**Figure S3.** Incidence rates for stable sexual partnership formation per 1,000 person‐years with an HIV‐positive, HIV‐negative or unknown serostatus partner by participant's own time‐varying HIV status and sex: (A) females and (B) males. Error bars represent 95% confidence intervals for the incidence rates of stable sexual partnership formation per 1,000 person‐years.
**Figure S4.** Estimated proportion of each type of the partnerships using the random impuation method for HIV seroconcverstion dates. Estimations are based on 200 realizations of the model.
