# Supplementary material for: HIV seroconcordance among heterosexual couples in rural KwaZulu‐Natal, South Africa: a population‐based analysis
Source: J Int AIDS Soc. 2020 Jan 8;23(1):e25432. doi: 10.1002/jia2.25432 (PMC6949466; doi:10.1002/jia2.25432)
Supplement: Supplementary file 1 — Data S1. Individual‐based microsimulation model description. Table S1. Estimated proportion of each type of the partnerships. Estimations are based on 10,000 realizations of the model. Table S2. Hazard ratios for stable sexual partnership formation with HIV‐negative partners among all participants. Table S3. Hazard ratios for stable sexual partnership formation with HIV‐positive partners among all participants. Table S4. Estimated proportion of each type of the partnerships using the random impuation method for HIV seroconversion dates. Estimations are based on 200 realizations of the model. Figure S1. Distribution of the proportion of HIV‐positive seroconcordant partnerships. Estimations are based on 10,000 realizations of the model. Figure S2. Estimated proportion of each type of the partnerships. Estimations are based on 10,000 realizations of the model. Figure S3. Incidence rates for stable sexual partnership formation per 1,000 person‐years with an HIV‐positive, HIV‐negative or unknown serostatus partner by participant's own time‐varying HIV status and sex: (A) females and (B) males. Error bars represent 95% confidence intervals for the incidence rates of stable sexual partnership formation per 1,000 person‐years. Figure S4. Estimated proportion of each type of the partnerships using the random impuation method for HIV seroconcverstion dates. Estimations are based on 200 realizations of the model. [file JIA2-23-e25432-s001.docx]

**Supplementary materials**

**Individual-based microsimulation model description**

We constructed a simple stochastic microsimulation model to assess the patterns of partnership formation between HIV-positive individuals under the assumption of complete random mixing between individuals in the population. We developed an individual-based model parameterized using variables derived from the study population (the Africa Health Research Institute longitudinal demographic surveillance cohort in rural KwaZulu-Natal, South Africa) such as total numbers of males and females, HIV prevalence per year, and total numbers of partnerships formed during the study period. The model is a yearly time-step in which HIV transmission is simulated per year based on the HIV prevalence for males and females, as well as partnership formation from 2003 to 2016.

First, 10,763 males and 18,939 females were included in the simulation. HIV serostatus was randomly assigned according to the HIV prevalence in a given year estimated from the study population. Next, male and female individuals were randomly selected and later a partnership was generated with probability *p*. This probability was estimated based on the total number of partnerships formed during the period of study (677 partnerships). Lastly, proportion of partnerships according to HIV serostatus of each couple was estimated. Final estimations were based on the average of 10,000 realizations of the model. The 95% confidence interval (CI) was estimated using the 2.5 and 97.5 percentile of the 10,000 realizations of the model. The model was developed using MATLAB R2016b.

Calculations are summarized in Table S1. Figure S1 illustrates the distribution of the proportion of HIV-positive seroconcordant partnerships estimated from the 10,000 realizations. Figure 2 illustrates the estimated proportion of each of the partnerships formed based on the HIV serostatus of each partner.

**Table S1.** Estimated proportion of each pattern of partnerships based on HIV status of each partner. Estimations are based on 10,000 realizations of the model

|  | **HIV-positive  seroconcordant (%)** | **HIV serodiscordant (%)** | **HIV-negative  seroconcordant (%)** | **HIV-positive unknown status**  **(%)** | | **HIV-negative Unknown status**  **(%)** |
| --- | --- | --- | --- | --- | --- | --- |
| **Mean** | 2.30 | 15.17 | 25.10 | 16.48 | 40.94 | |
| **95% CI-low** | 1.31 | 12.54 | 21.87 | 13.70 | 37.32 | |
| **95% CI-high** | 3.50 | 17.78 | 28.28 | 19.39 | 44.75 | |

**Figure S1**. Distribution of the proportion of HIV-positive seroconcordant partnerships estimated from the 10,000 realizations of the model

**Figure S2.** Estimated proportion of each of the partnerships formed based on the HIV serostatus of each partner

**Incidence rates for stable sexual partnership formation by sex**

Incidence rates for stable sexual partnership formation per 1,000 person-years with a HIV-positive, HIV-negative or unknown status partner by participant's own HIV status and sex are shown in Figure S3.

|  | **Participant’s HIV status** | | | | | | | | |
| --- | --- | --- | --- | --- | --- | --- | --- | --- | --- |
|  | HIV-negative (N=18,341) | | | |  | HIV-positive (N=11,368) | | | |
|  | Formation No. | PY | Incidence Rate/100 PY | 95% CI |  | Formation No. | PY | Incidence Rate/100 PY | 95% CI |
| **A) Female participants** |  |  |  |  |  |  |  |  |  |
| Partner's HIV Status |  |  |  |  |  |  |  |  |  |
| Negative | 59 | 57751 | 1.02 | (0.79, 1.32) |  | 36 | 48491 | 0.74 | (0.54, 1.03) |
| Positive | 13 |  | 0.23 | (0.13, 0.39) |  | 28 |  | 0.58 | (0.40, 0.84) |
| Unknown | 108 |  | 1.87 | (1.55, 2.26) |  | 122 |  | 2.52 | (2.11, 3.00) |
| **B) Male participants** |  |  |  |  |  |  |  |  |  |
| Partner's HIV Status |  |  |  |  |  |  |  |  |  |
| Negative | 72 | 33880 | 2.13 | (1.69, 2.68) |  | 17 | 14335 | 1.19 | (0.74, 1.91) |
| Positive | 18 |  | 0.53 | (0.33, 0.84) |  | 24 |  | 1.67 | (1.12, 2.50) |
| Unknown | 104 |  | 3.07 | (2.53, 3.72) |  | 76 |  | 5.30 | (4.23, 6.64) |

**Figure S3. Incidence rates for stable sexual partnership formation per 1,000 person-years with a HIV-positive, HIV-negative or unknown status partner by participant's own HIV status and sex: (A) females and (B) males.** Error bars represent 95% confidence interval for the incidence rates of stable sexual partnership formation per 1,000 person-years.

**Hazard ratios for stable sexual partnership formation with HIV-negative partners**

We estimated the hazard of stable sexual partnership formation with known HIV seronegative partners, where formation with known seropositive or unknown serostatus partners was fitted as the competing risks. Forming a sexual partnership with an HIV-negative partner was 1.47 (95% CI: 1.01-2.14) times higher in HIV-negative individuals than in HIV-positive individuals after adjusting for covariates (Table S2).

| **Table S2. Hazard ratios for stable sexual partnership formation with HIV-negative partners among all participants** | | |
| --- | --- | --- |
|  | **Model 5** | **Model 6** |
| **Characteristics** | Hazard Ratio  (95% CI) | Adjusted Hazard Ratio (95% CI) |
| **HIV status** |  |  |
| Negative vs. Positive | **1.39 (1.00-1.93)** | **1.47 (1.01-2.14)** |
| **HIV prevalence in the opposite sex (per 10% increase)** |  | 1.01 (1.00-1.02) |
| **ART coverage (per 10% increase)** |  | 0.67 (0.58-0.79) |
| **HIV prevalence in the local area (per 10% increase)** |  | **1.39 (1.00-1.92)** |
| **Age at baseline (years)** |  |  |
| ≥30 vs <30 |  | 1.02 (0.66-1.60) |
| **Socioeconomic status (Household Asset)** |  |  |
| Poorest or Poor |  | **1.76 (1.11-2.81)** |
| Medium |  | 0.98 (0.55-1.72) |
| Rich or Richest |  | Ref |
| **Education** |  |  |
| Secondary+ (≥ grade 8) |  | 0.92 (0.49-1.73) |
| Primary (grade 1-7) |  | 1.13 (0.59-2.15) |
| No formal education |  | Ref |
| **Area of residence** |  |  |
| Peri-urban or Urban vs Rural |  | 0.92 (0.62-1.34) |
| **Ever reporting to have >1 partner in the last 12 months** |  |  |
| Yes vs. No |  | 1.11 (0.69-1.79) |

†The model was adjusted for all other variables shown in the column.

§Abbreviations: HIV, Human Immunodeficiency Virus; ART, Antiretroviral Therapy

**Sensitivity analysis of handling missing covariates using multiple imputation**

The five covariates (ART coverage, HIV prevalence in the local area, household asset, area of residence, and ever reporting to have >1 partner in the last 12 months) were imputed using multiple imputation by chained equations (MICE) in STATA 15 [1,2]. The continuous variables (ART coverage and HIV prevalence in the local area) were imputed using linear regression while the binary variables (area of residence and ever reporting to have >1 partner in the last 12 months) were imputed using logistic regression and the ordered categorical variable (household asset) using ordered logistic regression. The cox regression model was fitted over the imputed datasets to estimate the hazard of stable sexual partnership formation with known HIV seronegative partners. We found that there were no significant changes in the model estimates when missing covariates were adjusted using multiple imputation (Table S3).

**Table S3. Hazard ratios for stable sexual partnership formation with HIV-positive partners among all participants**

|  | **Model 7** |
| --- | --- |
| **Characteristics** | Adjusted Hazard Ratio  (95% CI) |
| **HIV status** |  |
| Positive vs negative | **2.33 (1.42, 3.84)** |
| **HIV prevalence of the opposite sex (per 10% increase)** | 1.00 (0.99, 1.02) |
| **ART coverage (per 10% increase)** | 0.86 (0.72, 1.02) |
| **HIV prevalence in the local area (per 10% increase)** | 1.11 (0.69, 1.79) |
| **Age at baseline (years)** |  |
| ≥30 vs <30 | **1.79 (1.02, 3.11)** |
| **Socioeconomic status (Household Asset)** |  |
| Poorest or Poor | **2.24 (1.29, 3.89)** |
| Medium | 1.35 (0.68, 2.68) |
| Rich or Richest | Ref |
| **Education** |  |
| Secondary or Tertiary (≥ grade 8) | 0.82 (0.37, 1.84) |
| Primary (grade 1-7) | 1.16 (0.52, 2.62) |
| No formal education | Ref |
| **Area of residence** |  |
| Peri-urban or Urban | 1.62 (0.89, 2.97) |
| Rural | Ref |
| **Ever reporting to have >1 partner in the last 12 months** |  |
| Yes vs. No | 1.51 (0.78, 2.93) |

†The model was adjusted for all other variables shown in the column.

§Abbreviations: HIV, Human Immunodeficiency Virus; ART, Antiretroviral Therapy

**HIV seroconcordance and serodiscordance using the imputed dates of seroconversion from the uniform random distribution**

We performed sensitivity analysis where we imputed the date of seroconversion from random uniform distribution and calculated the proportion of HIV seroconcordance and serodiscordance among the 677 newly formed stable sexual partnerships based on the average of 200 simulations. The 95% confidence interval (CI) was estimated using the 2.5 and 97.5 percentile of the 200 simulations.

Calculations are summarized in Table S4. Figure S4 illustrates the distribution of the proportion of each of the partnerships estimated from the 200 simulations.

**Table S4.** Estimated proportion of each pattern of partnerships based on HIV status of each partner. Estimations are based on 200 simulations

|  | **HIV-positive  seroconcordant (%)** | **HIV serodiscordant (%)** | **HIV-negative  seroconcordant (%)** | **HIV-positive unknown status**  **(%)** | | **HIV-negative Unknown status**  **(%)** |
| --- | --- | --- | --- | --- | --- | --- |
| **Mean** | 8.25 | 11.73 | 19.45 | 30.12 | 30.44 | |
| **95% CI-low** | 7.83 | 10.78 | 18.91 | 29.10 | 29.54 | |
| **95% CI-high** | 8.71 | 12.56 | 20.09 | 31.02 | 31.46 | |

**Figure S4.** Estimated proportion of each of the partnerships formed based on the HIV serostatus of each partner

**MATLAB script for simulation model**

%#### SEROCONCORDANCE MODEL August 2018 ######

%####Authors: Hae-Young Kim, Guy Harling, Alain Vandormael, Andrew Tomita, Diego F. Cuadros, Till Bärnighausen, Frank Tanser ######

%### SIMULATION PARAMENTERS ####

Males_number=10763; %number of males in the simulation

Females_number =18939;% number of females in the simulation

prob_p = 0.00256;%probability of partnership formation, I used this number to approach the actual number of partnerships observed

Partners = Females_number*prob_p;%Calculates how many partnerships are formed

Prev_M = [0.105 0.128 0.141 0.139 0.138 0.142 0.169 0.163 0.169 0.180 0.204 0.220 0.253 0.317]; %time-series HIV prevalence in males from 2003 to 2016

Prev_F = [0.190 0.236 0.248 0.257 0.211 0.231 0.253 0.271 0.288 0.298 0.320 0.341 0.375 0.429]; %time-series HIV prevalence in females from 2003 to 2016

years = length(Prev_M);%estimate how many years are simulated based on the HIV prevalence input

N_simulations = 10000;%number of realizations of the model

Results =[];%matrix for storing the results of each simulation

%###### SIMULATION LOOP ########

for S = 1:N_simulations

store_partner=[];%matrix that store the information of each partnership formed

Males = [zeros(Males_number,3)]; %matrix that store information for males (HIV status and index of the couple

Females= [zeros(Females_number,3)];%same for females

for n = 1:years %simulation of the time-series

%first randomly include the HIV-infected population for males and females according to the HIV prevalence for each

prevM=0;

while prevM < Prev_M(n)

M_HIV = floor(1+rand*(Males_number-1));

if Males(M_HIV,1)==0

Males(M_HIV,1) = 1;

end

prevM = sum(Males(:,1))/Males_number;

end

prevF=0;

while prevF < Prev_F(n)

F_HIV = floor(1+rand*(Females_number-1));

if Females(F_HIV,1)==0

Females(F_HIV,1) = 1;

end

prevF = sum(Females(:,1))/Females_number;

end

%second, partnership formation assuming complete random mixing

p=0;

while p < Partners

fem_rand = floor(1+rand*(Females_number-1));%random selection of females

male_rand = floor(1+rand*(Males_number-1));%random selection of males

if Females(fem_rand,2) == 0 && Males(male_rand,2)==0

Females(fem_rand,2)=1;

Females(fem_rand,3)=male_rand;

Males(male_rand,2)=1;

Males(male_rand,3)=fem_rand;

store_partner =[store_partner;Females(fem_rand,1) Males(male_rand,1)];%partners are stored

p=p+1;

end

end

end

%#serosorting estimations

count_concordant = 0;

count_discordant=0;

count_negative = 0;

count_uknown_positive = 0;

count_uknown_negative = 0;

total_partner =length(store_partner(:,1));%double check how many partnerships were formed

for f = 1:total_partner %for each partnership

if rand < 0.426 %randomly select partnerships with known HIV status for both partners

if store_partner(f,1)==1 && store_partner(f,2)==1 %HIV positive concordant

count_concordant = count_concordant+1;

elseif store_partner(f,1)==1 && store_partner(f,2)==0 %HIV discordant

count_discordant=count_discordant +1;

elseif store_partner(f,1)==0 && store_partner(f,2)==1 %HIV discordant

count_discordant=count_discordant +1;

elseif store_partner(f,1)==0 && store_partner(f,2)==0 %HIV negative concordant

count_negative=count_negative +1;

end

else %Partners with unknown HIV status

if store_partner(f,1)==1

count_uknown_positive = count_uknown_positive + 1;

elseif store_partner(f,1)==0

count_uknown_negative = count_uknown_negative + 1;

end

end

end

%#Percentage calculation for each subpopulation

Positive_concordant=(count_concordant/total_partner)*100;%HIV positive concordant

Discordant=(count_discordant/total_partner)*100;%HIV discordant

Negative_concordant=(count_negative/total_partner)*100;%HIV negative concordant

HIV_positive_uknown =(count_uknown_positive/total_partner)*100;%Partner's HIV status unknown

HIV_negative_unknown =(count_uknown_negative/total_partner)*100;

Results(S,:) =[Positive_concordant Discordant Negative_concordant HIV_positive_uknown HIV_negative_unknown];%stores the results of each realization of the model

end %end of the simulations

%###### SUMMARY STATISTICS ########

Tot_percent = mean(Results);%calculates the average of the estimations from the total number of realizations

CI = [prctile(Results, 2.5); prctile(Results, 97.5)];%calculates the confidence interval for each estimation

text = [string('Positive_concordant') ,string('Discordant'), string('Negative_concordant'), string('HIV_positive_unknown'), string('HIV_positive_unknown')];

Report_results1 = [string('_'); string('Average'); string('CI_low'); string('CI_High')];

Report_results2 = [text;Tot_percent;CI];

Final_Report= [Report_results1 Report_results2] %final report

**References**

1. Royston P, White I. Multiple Imputation by Chained Equations (MICE): Implementation in Stata. J Stat Softw. 2011;45(4):1–20.

2. White IR, Royston P, Wood AM. Multiple imputation using chained equations: Issues and guidance for practice. Stat Med. 2011;30(4):377–99.
